# Video-assisted thoracoscopic surgery for intralobar pulmonary sequestration

**DOI:** 10.1186/1757-1626-1-269

**Published:** 2008-10-24

**Authors:** Dimitrios Avgerinos, Angelo Reyes, Eduardo Plantilla, Merab Krikhely

**Affiliations:** 1 Department of Surgery, Beth Israel Medical Center, Albert Einstein College of Medicine, First Avenue at 16th Street, New York, New York, USA; 2 Division of Thoracic Surgery Surgery, Beth Israel Medical Center, Albert Einstein College of Medicine, First Avenue at 16th Street, New York, New York, USA

## Abstract

**Introduction:**

Intralobar pulmonary sequestration is a rare congenital abnormality of the lower respiratory system, which becomes symptomatic early in life. Standard treatment consists of wedge resection or lobectomy through a thoracotomy.

**Case presentation:**

We report on an unusual case of a 36-year-old female patient with intralobar pulmonary sequestration on the right lower lobe, which was treated with video-assisted thoracic surgery. The case is presented along with literature review.

**Conclusion:**

VATS wedge resection is a great alternative to the traditional thoracotomy for the treatment of intralobar pulmonary sequestrations.

## Introduction

Pulmonary sequestration is a rare congenital abnormality of the lower respiratory system, consisting of about 0.15–6.4% of all pulmonary malformations [1]. The standard treatment of this entity used to be either wedge resection or lobectomy through a thoracotomy, after identification and ligation of the aberrant artery of the mass. However, recent advancements in technology have permitted a more minimally invasive approach by using video-assisted thoracoscopic surgery (VATS). This report illustrates a case in which a VATS wedge resection was employed successfully to treat intralobar pulmonary sequestration in an adult. A Medline search of the English literature revealed that this is only the second published case in the United States of intralobar pulmonary sequestration in an adult that was treated with VATS.

## Case presentation

**Figure 1:**
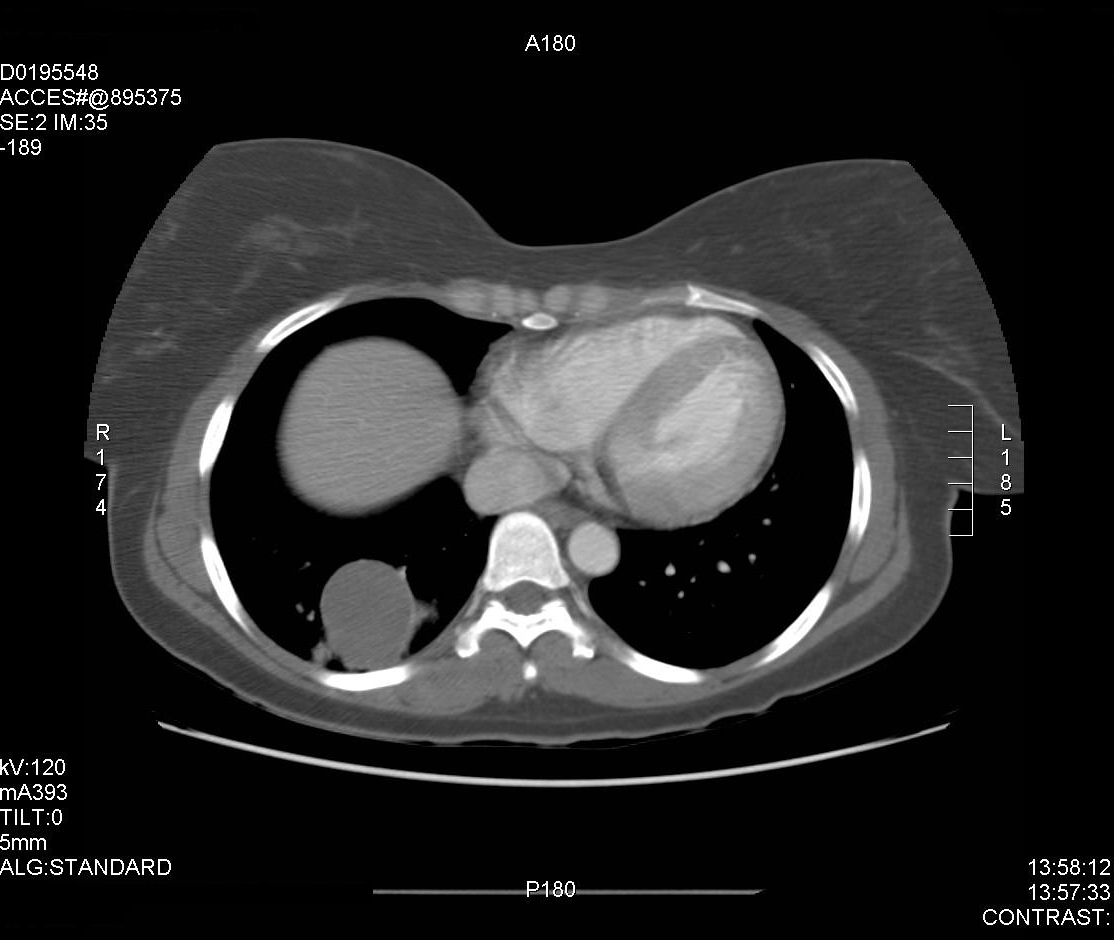
CT scan of the lower lungs/upper abdomen indicating the aberrant feeder artery originating from the descending aorta to the area of the right lower lobe mass.

**Figure 2:**
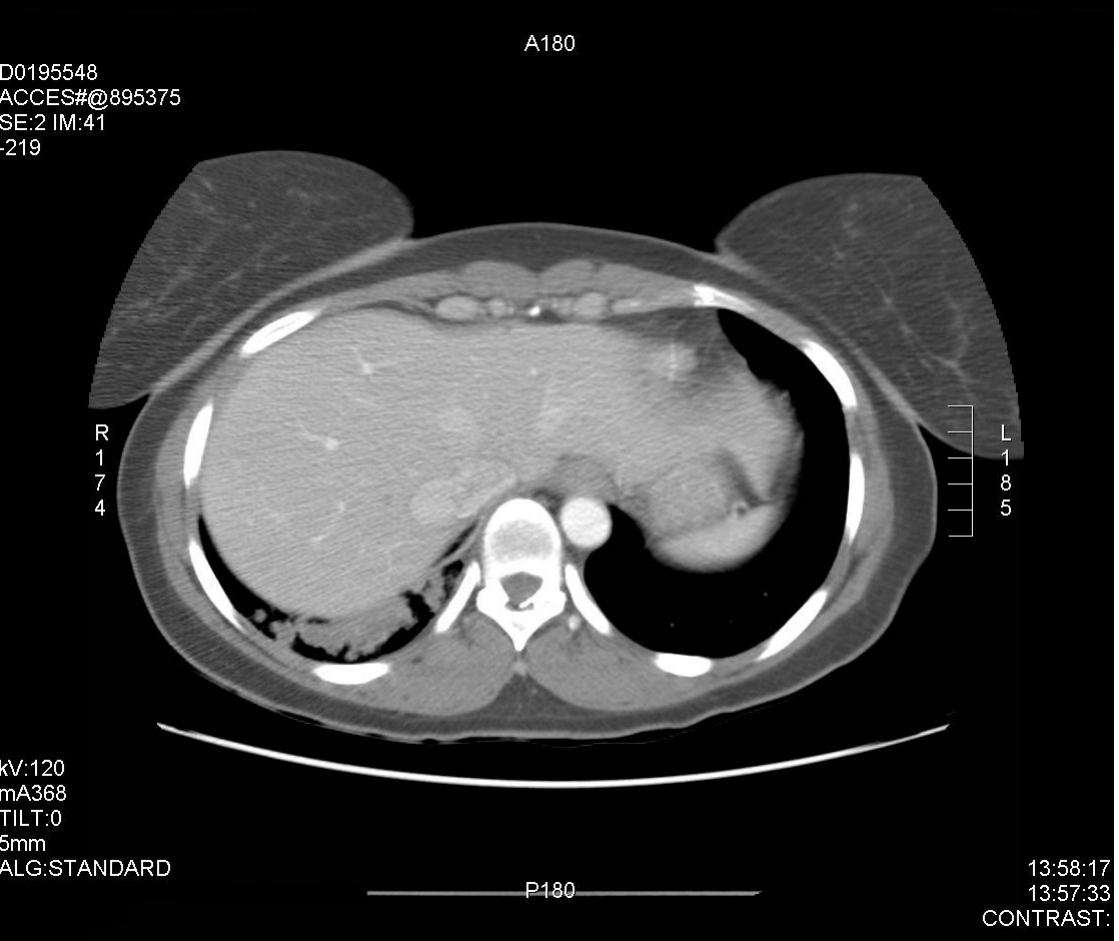
CT scan of the lower lungs/upper abdomen indicating the aberrant feeder artery originating from the descending aorta to the area of the right lower lobe mass.

A thirty-six year old female was referred to our hospital with a 16-year history of right rib pain and recurrent lower respiratory system infections. The patient had multiple computed tomography scans in the past, but the diagnosis was never established with success as intravenous contrast was never used. The patient had no other symptoms at presentation and no abnormality was found on physical examination. The only abnormal laboratory value was a slight increase in the white blood cell count, which was 14,000 cells/mm^3^. A chest radiograph was obtained and was normal. The diagnosis was established with a computed tomography (CT) scan of the chest with intravenous contrast. The test revealed a mass in the right lower lobe (measuring 4.5 × 3.8 × 2.9 cm). An aberrant artery (3 mm in diameter) arose from the abdominal aorta and flowed into the lesion (see Figures 1 and 2). The patient was diagnosed with intralobar pulmonary sequestration and surgical exploration and treatment with video-assisted thoracoscopic surgery was planned.

**Figure 3:**
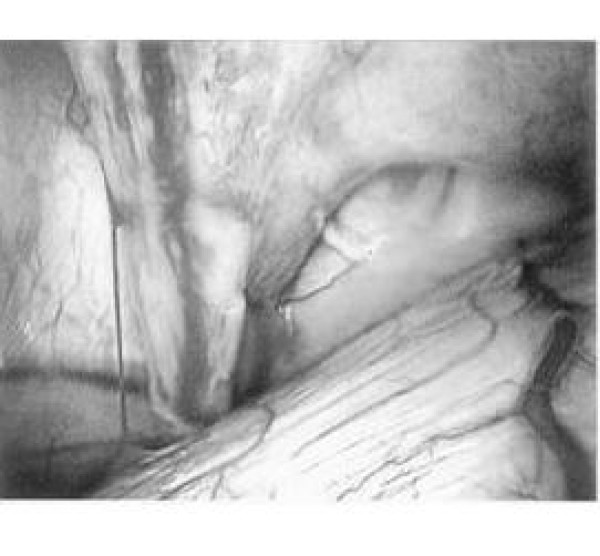
Intraoperative thoracoscopic picture of the pulmonary sequestration showing the aberrant artery originating from the infradiaphragmatic aorta.

After getting the appropriate consent for the operation and administering general anesthesia and intubation, the patient was placed in a full left lateral decubitus position with the table flexed at 30-degrees at the level between the nipples and the umbilicus in order to have better exposure of the right intercostals spaces. A 10 mm zero-degree thoracoscope was inserted in the right pleural cavity through a port site placed in the sixth intercostal space on the midaxillary line. Two additional port sites were placed in the fifth intercostal space on the posterior and anterior midaxillary line, respectively. The port sites were chosen with a possible thoracotomy in mind. The VATS exploration revealed immediately a mass in the base of the right lung. Upon retraction of the mass, an aberrant artery coming through the diaphragm and leading into the mass was identified (see Figure 3). The artery was carefully dissected from the surrounding tissues and divided using an endothoracic stapling device (Endopath 45, Ethicon Endo-Surgery, Inc., Cincinnati, Ohio). The same device was used in order to perform a tailored wedge resection of the right lower lobe, removing this way the pulmonary sequestration. Inspection of the rest of the lung revealed normal pulmonary parenchyma.

After closing the port sites and inserting a chest tube, the patient was extubated and was transferred to the surgical intensive care unit for observation. Her recovery was uneventful and the chest tube was put on water seal the next morning and removed on post-operative day 2. The patient was discharged the third post-operative day and until today, about five months after the operation, remains in excellent condition. Final pathology revealed intralobar pulmonary sequestration.

## Discussion

Pulmonary sequestration is a rare lesion of the lung parenchyma of unknown etiology that lacks normal connection with the tracheobronchial tree and has a blood supply directly from the descending aorta [2]. There are two basic types: extralobar (25% of all cases), which have their own pleura, and intralobar (75% of all cases), which are surrounded by visceral pleura [3,4]. Patients with pulmonary sequestration tend to present early in life with symptoms of recurrent infections of the lower respiratory system. As a result, they undergo definitive operative treatment at a young age. Work-up to investigate the etiology of the repeated episodes of pneumonia should include a thorough history and physical investigation, usual labs (complete blood count and basic metabolic panel), chest x-ray, and, ultimately, CT scan of the chest with intravenous contrast. The last modality makes the diagnosis and is regarded as the imaging test of choice for pulmonary sequestration.

The definitive treatment of every patient with pulmonary sequestration, both intralobar and extralobar, is surgical resection of the mass. Traditionally, the open approach through posterolateral thoracotomy has been employed, as it offers adequate exposure. Because pulmonary sequestration is a benign disease, it is believed that partial lung resection is more appropriate than lobectomy [5], especially if the mass is confined within a lung segment [6]. Special attention needs to be paid to the aberrant artery, since its path through the diaphragm needs to be identified before it is ligated and divided. The advent of minimally invasive techniques made VATS gain special role in the treatment of this pathologic entity. VATS offers an important alternative to the open approach for pulmonary sequestration, with minimum surgical trauma morbidity, post-operative pain, and hospitalization. It is characteristic that our patient was discharged home on the second post-operative day in an excellent condition. Our experience has shown that patient undergoing thoracotomy for the same disease usually stay in the hospital from 3 to 5 days, mainly for surgical site pain management. Also, VATS has been shown to better preserve lung function during the recovery period [7,8]. An important note, if VATS is employed for the resection of intralobar pulmonary sequestration, is that extensive pre-operative work-up is needed for the identification of the aberrant artery. Its connection to the thoracic or abdominal aorta can be identified with the use of computed tomography (CT) with intravenous contrast or even CT angiography.

## Conclusion

We believe that VATS wedge resection is a great alternative to the traditional thoracotomy for the treatment of intralobar pulmonary sequestrations.

## Consent

Written informed consent was obtained from the patient for publication of this case report and accompanying images. A copy of the written consent is available for review by the Editor-in-Chief of this journal.

## Competing interests

The authors declare that they have no competing interests.

## Authors' contributions

DA and AR analyzed and interpreted the patient data. EP performed the literature review, and was a major contributor in writing the manuscript. AR and MK performed the final editing of the manuscript. All authors read and approved the final manuscript.
